# Genome sequence of a virulent and hypermucoviscous-like *Klebsiella michiganensis* clinical isolate

**DOI:** 10.1186/s13104-023-06603-9

**Published:** 2023-11-14

**Authors:** Alejandro Alvarado-Delgado, Nadia Rodríguez-Medina, Alejandro Sánchez-Pérez, Elsa María Tamayo-Legorreta, Jackeline Cerón-Lopez, Rayo Morfin-Otero, Ulises Garza-Ramos

**Affiliations:** 1 Instituto Nacional de Salud Pública (INSP), Centro de Investigación Sobre Enfermedades Infecciosas (CISEI), Grupo de Investigación y Docencia en Resistencia Antimicrobiana (GID-RAM), Cuernavaca, Morelos México; 2 Hospital Civil de Guadalajara Fray Antonio Alcalde, Universidad de Guadalajara, Guadalajara, Jalisco Mexico

**Keywords:** Hypermucoviscous phenotype, String test, Capsule, Virulence, *Klebsiella michiganensis*

## Abstract

**Objectives:**

The hypermucoviscous-like phenotype has been described in *Klebsiella pneumoniae* species complex (KpSC) and was described as a contributor of increased virulence. This study described the characterization and whole-genome sequencing of an antibiotic susceptible and hypermucoviscous-like *Klebsiella michiganensis* 9273 clinical isolate.

**Data description:**

Here, we report the genome sequence of a *K*. *michiganensis* clinical isolate obtained from a urinary tract infection exhibiting the hypermucoviscous-like phenotype. The draft genome sequence consisted of 145 contigs and ~ 6.6 Mb genome size. The annotation revealed 6648 coding DNA sequences and 56 tRNA genes. The strain belongs to the sequence type (ST) 50, and the OXY-1 beta-lactam resistance gene, *aph(3′)-Ia* gene for aminoglycoside resistance and multidrug efflux pumps were identified. The *fyuA* siderophore receptor of yersiniabactin siderophore was identified. Increased virulence was observed *in Galleria mellonella* larvae model and increased capsule production was determined by uronic acid quantification. The clinical implications of this phenotype are unknown, but the patient outcome might worsen compared to susceptible- or MDR-classical *K. michiganensis* isolates.

**Supplementary Information:**

The online version contains supplementary material available at 10.1186/s13104-023-06603-9.

## Objective

The hypermucoviscous phenotype and hypercapsule production are driven by the *rmpADC* operon identified in *Klebsiella pneumoniae* [1]. There are several reports of hypermucoviscous KpSC isolates that do not carry the *rmpADC* operon; hereafter this phenotype is refer to as hypermucoviscous-like. This phenotype has been  described in *K. pneumoniae *[2], *K. quasipneumoniae* subsp. *similipneumoniae* [3] and *K. variicola* [4] but rarely seen outside the KpSC. It has been demonstrated that the hypermucoviscous-like phenotype promotes virulence, so those isolates displaying this phenotype are likely to increase its virulence [4]. The *Klebsiella oxytoca* complex (KoxSC) comprise nine species in which *K. michiganensis* is a member*.* Precise species identification requires genome-based analysis thus misidentification is likely to occur by phenotypic, biochemical test or MALDI-TOF [5].

The prevalence and type of infections caused by *K. michiganensis* is largely unknown but the species of the KoxSC are opportunistic pathogens that are associated with hemorrhagic colitis, urinary tract infections and bacteremia, and occasionally outbreaks in immunocompromised patients or those requiring intensive care [5]. *K. michiganensis* may acquire extended-spectrum β-lactamases (ESBL), carbapenemases and colistin resistance [6, 7]. To date, no hypervirulent or hypermucoviscous-like *K*. *michiganensis* strains has been described. In this study, the semi-quantitative string test [8] was applied to a collection of 30 presumptive *K. oxytoca* isolates resulting one isolate positive to this test which was further characterized.

### Data description

The *K. michiganensis* 9273 isolate was obtained from urinary tract infection at the Hospital Civil de Guadalajara, Mexico in 2015. First, the species identification was performed at the hospital using MicroScan and resulted in *K*. *oxytoca*. The strain was subjected to disk-diffusion-method, accordingly to CLSI (2022) guidelines [9], to determine its antimicrobial susceptibility profile. It showed resistance to ampicillin (256 mg/ml) and susceptibility to ceftazidime, cefotaxime, imipenem, amikacin, gentamicin, nalidixic acid, ciprofloxacin, gentamicin, sulfamethoxazole plus trimethoprim and tetracycline, which characterize a non-MDR profile. The string test was evaluated in Mac Conkey agar plates and revealed a 4 cm string long. The plasmid profile was investigated by alkaline lysis protocol using the *E. coli* NCTC 50192 strain, which contains plasmids of 154-,66-, 48-, and 7-kb as a molecular size marker [10]. The *K*. *michiganensis* 9273 isolate possess three plasmids of ~ 190-, 160-, and 90-kb (Additional file 1: Fig. S1).

Total genomic DNA from the 9273-isolate was extracted using 5 ml of an overnight culture then purified using the DNeasy Kit (Qiagen, Germany). In 2015, the whole-genome sequence was generated using pyrosequencing methodology with the 454 Roche FLX Titanium platform. Reads (99.9% above Q40) longer than 500 bp were used for de novo assembly with the CLC Genomics Workbench version 4.0 (CLC bio). The total sequence data are 295,456 reads with 30- to 943-bp length range and a total of 145 contigs, with an estimated genome size of 6,632,597 bp with 20X coverage. The genome sequence was subjected to average nucleotide identity (ANI) analysis against reference genomes of the nine members of the KoxSC. The ANI values of *K. oxytoca* (GCA_003812925.1), *K. grimontii* (GCA_900200035), *K. huaxiensis* (GCA_003261575)*, K. pasteurii* (GCA_018139045.1)*, K. spallanzanii* (GCA_901563875.1)*,* taxon 1 (QJJG00000000), taxon 2 (CP046115) and taxon 3 (CP055481) were below the cutoff point for species distinction (87.3% to 91.7%) but 99.24% ANI value with *K. michiganensis* (GCA_015139575.1) was observed*.* The Whole Genome Sequencing project was deposited at DDBJ/ENA/GenBank under the accession number JAVFHI000000000.

MLST typing tool determined the sequence type (ST) 50 (https://cge.cbs.dtu.dk/services/MLST/). This ST50 did not correspond to globally expanding beta-lactam resistant sequence type [11]. Carriage of antimicrobial resistance genes was determined by ResFinder-4.1 (https://cge.cbs.dtu.dk/services/ResFinder/). The OXY-1 β-lactam resistance gene was identified which represents the phylogenetic group Ko1. In addition, the *aph(3')-Ia* gene for aminoglycoside resistance and multidrug efflux pumps (*acrD*, *acrB*, *mdtB*, *mdtC*, *bepE*, *msbA*, and *emrB* genes) were identified. Lastly, virulence genes *mrk*, *kfu, fyuA* (siderophore receptor of yersiniabactin) were found.

Plasmids with incompatibility groups repA, IncFIB_K_ and IncFII were detected by PlasmidFinder-V2.1 (https://cge.food.dtu.dk/services/PlasmidFinder/). The hypermucoviscous-like phenotype in *K. variicola* has been linked with the acquisition of an IncFIB_K_-plasmid (pKV8917) which conferred the highly viscous phenotype. The pKV8917 self-transmissible plasmid has the potential to disseminate to other *Klebsiella* species [4]. Thus, additional studies are required to determine whether there is a plasmid-borne or chromosomal mechanism in *K. michiganensis* 9273*.*

The relationship between resistance genes and mobile genetic element were predicted using Mobile Element Finder (https://cge.food.dtu.dk/services/MobileElementFinder/) showing that *aph(3′)-Ia* and *fyuA* belonged to the same contig, suggesting a putative co-harboring location.

**Fig. 1:**
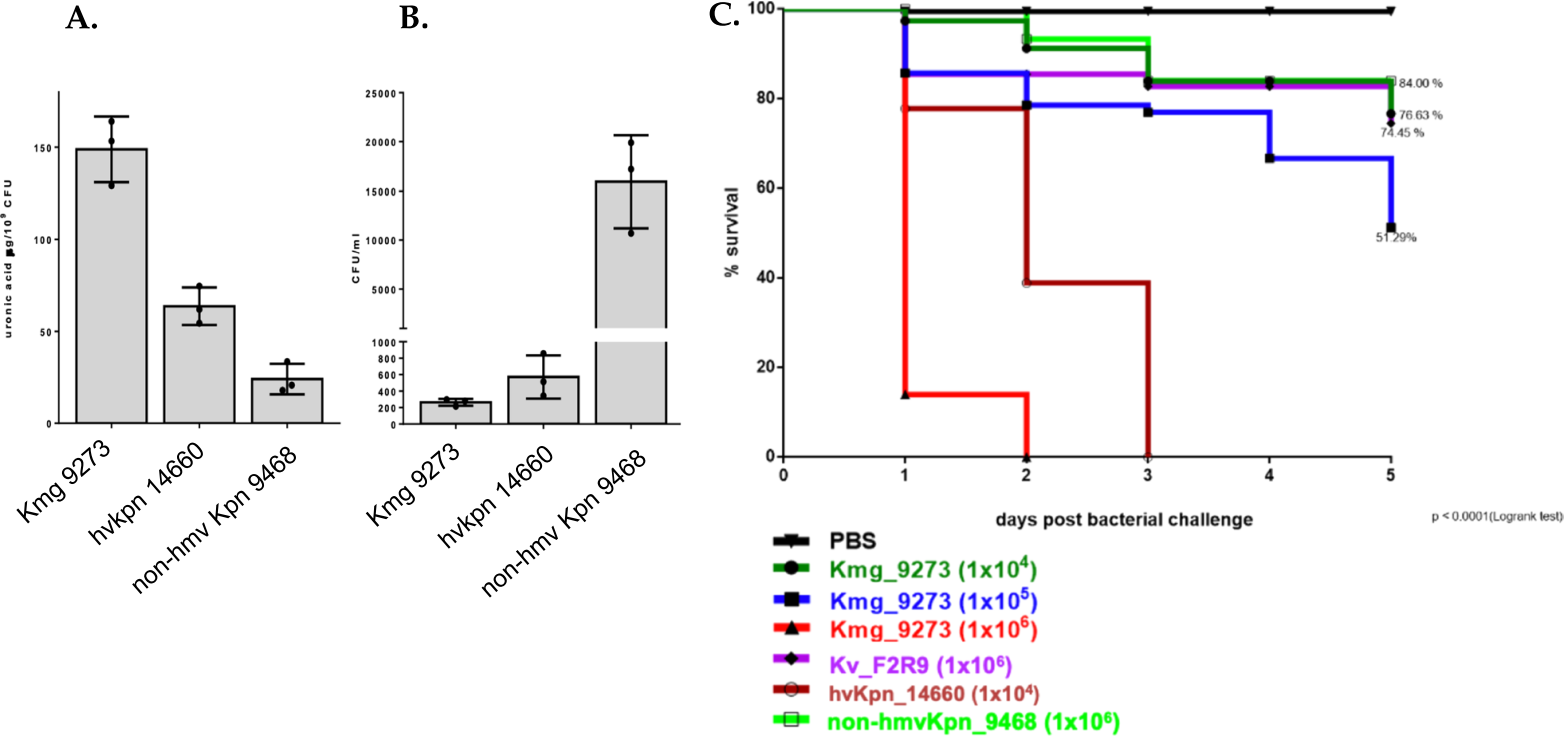
Assays for determining the capsule-associated virulence phenotype in *K*. *michiganensis* 9273. **A** Quantification of uronic acid and **B** Phagocytosis assay. We included one hypervirulent *K. pneumoniae* (14660, ST86-K2) and one classical and non-hypermucoviscous *K. pneumoniae* (9468) as reference strains for capsule production and phagocytosis resistance assays. **C** Kaplan–Meier survival curves of infected *G. mellonella* larvae with *K. michiganensis*  isolate 9273 at three doses 1 × 10^4^, 1 × 10^5^ and 1 × 10^6^ CFUs. *K. variicola* F2R9 (10^6^ CFUs) [12], *K. pneumoniae* 9468 (10^6^ CFUs) and hypervirulent *K. pneumoniae* 14660 (10^4^ CFUs) were used respectively as nonlethal and lethal doses control. Statistical analysis was carried out based on one way ANOVA (**A** and **B**), long rank (Mantel-Cox) and Chi-square test (**C**); *p* < 0.0001. Uronic acid quantification and phagocytosis assay are presented as the mean ± standard deviation of three independent experiments. *Kmg* *K. michiganensis*, *hvKpn* hypervirulent *K. pneumoniae,* *non-hmv Kpn* non-hypermucoviscous *K. pneumoniae,* *PBS* Phosphate-buffered saline

Quantification of capsule by means of uronic acid measurement [4] revealed that the hypermucoviscous-like *K. michiganensis* isolate 9273 produced more capsule (148 µg/10^9^ CFUs) than non-hypermucoviscous (24 µg/10^9^ CFUs) and hypervirulent *K. pneumoniae* (63.6 µg/10^9^ CFUs) isolates reported in previous studies (Fig. 1A) [12]. Phagocytosis assay [4] showed that the 9273-isolate was less phagocyted in comparison to a non-hypermucoviscous isolate (Fig. 1B). In addition, we performed serum killing assay using human serum obtained from healthy volunteers [4] and was used to challenge ~ 10^6^ CFUs of the 9273 isolate. This assay was performed in triplicate and revealed a serum resistant type.

Finally, infection assays were realized according to Sugeçti [13]. *G. mellonella* larvae were acquired from Petmmal company (México). Briefly, bacterial cultures of *K. michiganensis* 9273 were prepared with 10^4^, 10^5^ and 10^6^ CFU per 20 µl in PBS 1X. Seven-instar larvae of *G. mellonella* were chilled on ice for 5 min and surface sterilized in 95% ethanol. Then, 20 µl of each culture was injected into the hemocoel of each *G. mellonella* larvae with a hamilton syringe. Larvae were examined every 24 h and were scored as dead when they were melanized or unresponsive to touch.

We inoculated 10^6^ CFUs of classical *K. variicola* F2R9 [14] and *K. pneumoniae* 9468 as nonlethal controls and 10^4^ CFUs of the hypervirulent *K. pneumoniae* 14660 as lethal control. All experiments used 40 larvae per treatment. After 5 days post bacterial challenge, similar survival percentages were obtained for *K. michiganensis* 9273 (84%, 10^4^ CFUs), *K. variicola* F2R9 (76.63%, 10^6^ CFUs) and *K. pneumoniae* 9468 (74.45%, 10^6^ CFUs). However, inoculation of 10^5^ and 10^6^ CFUs of *K. michiganensis* 9273 resulted in 50% and 0% survival, respectively (Fig. 1C). Similarly, 0% survival for hypervirulent *K. pneumoniae* 14660 (10^4^) was observed after 3 days (Fig. 1C). Taken together, these results prove that *K. michiganensis* 9273 isolate is virulent, and this effect may be linked with its capsule-associated phenotype.

## Limitations

Unlike the fact that the hypermucoviscous-like phenotype in *K*. *variicola* was determined to be plasmid-mediated, the present work did not address the genetic basis for this phenotype.

### Supplementary Information

**Additional file 1: Figure S1.** Plasmid profile of the hypermucoviscous and virulent K. michiganensis 9273. Lines: 1, E. coli 50192 was used as molecular size maker; 2, Hypervirulent K. pneumoniae 14660, 3, the non-hypermucoviscous K. pneumoniae 9458 (absent of plasmids) and 4, Hypervirulent-hypermucoviscous K. michiganensis 9273. *Chr* Bacterial chromosome.

## Data Availability

The data described in this Data note can be freely and openly accessed under the Accession Number: JAVFHI000000000.
