# Supplementary material for: Genome sequence of a virulent and hypermucoviscous-like Klebsiella michiganensis clinical isolate
Source: BMC Res Notes. 2023 Nov 14;16:334. doi: 10.1186/s13104-023-06603-9 (PMC10647098; doi:10.1186/s13104-023-06603-9)
Supplement: Supplementary file 1 — Additional file 1: Figure S1. Plasmid profile of the hypermucoviscous and virulent K. michiganensis 9273. Lines: 1, E. coli 50192 was used as molecular size maker; 2, Hypervirulent K. pneumoniae 14660, 3, the non-hypermucoviscous K. pneumoniae 9458 (absent of plasmids) and 4, Hypervirulent-hypermucoviscous K. michiganensis 9273. Chr Bacterial chromosome. [file 13104_2023_6603_MOESM1_ESM.pptx]

## Slide 1
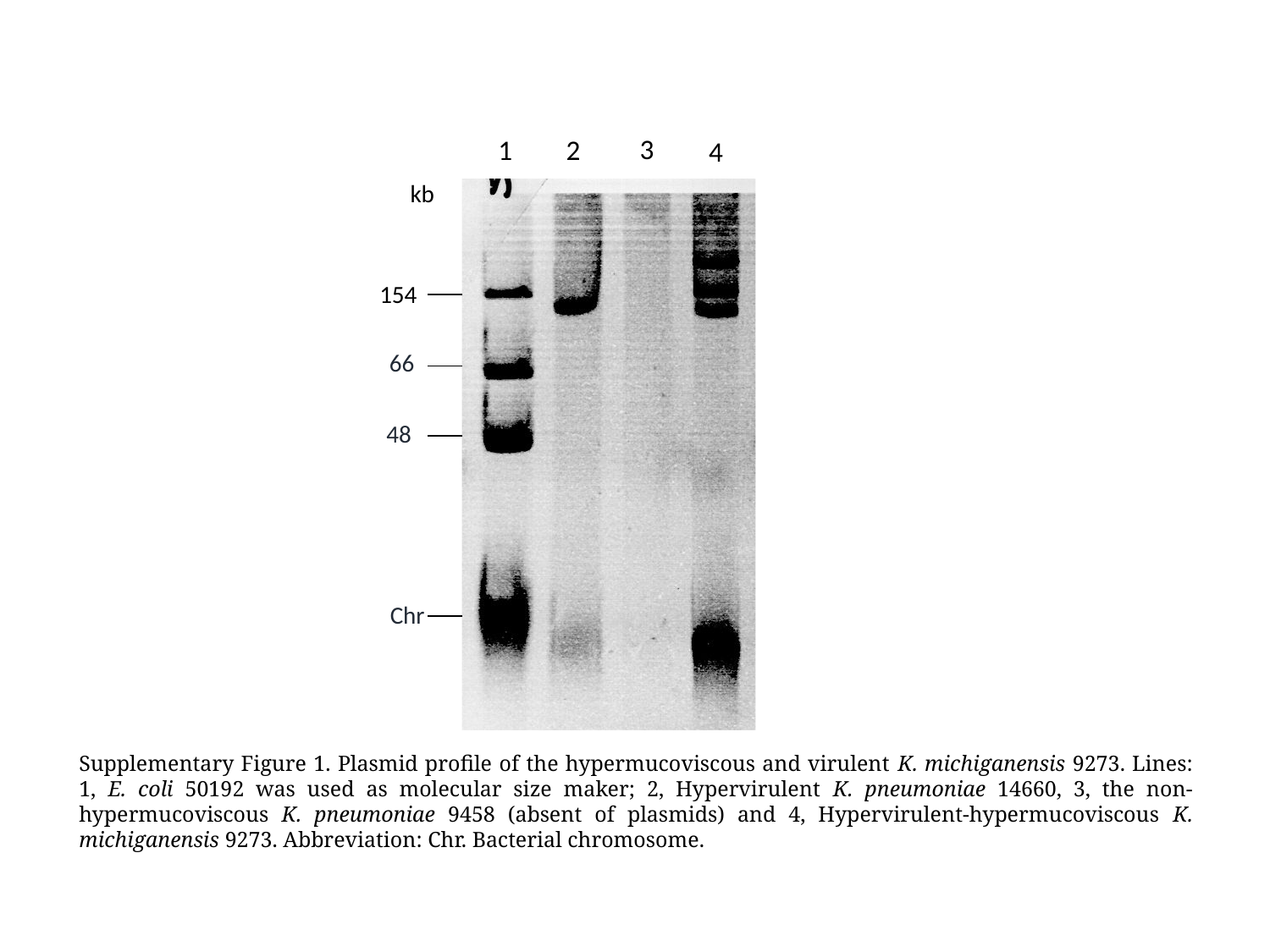

3
1
2
4
kb
154
66
48
Chr
Supplementary Figure 1. Plasmid profile of the hypermucoviscous and virulent K. michiganensis 9273. Lines: 1, E. coli 50192 was used as molecular size maker; 2, Hypervirulent K. pneumoniae 14660, 3, the non-hypermucoviscous K. pneumoniae 9458 (absent of plasmids) and 4, Hypervirulent-hypermucoviscous K. michiganensis 9273. Abbreviation: Chr. Bacterial chromosome.
